# Multiple Independent Oscillatory Networks in the Degenerating Retina

**DOI:** 10.3389/fncel.2015.00444

**Published:** 2015-11-09

**Authors:** Thomas Euler, Timm Schubert

**Affiliations:** ^1^Werner Reichardt Centre for Integrative Neuroscience (CIN)/Institute for Ophathalmic Research, University of TübingenTübingen, Germany; ^2^Bernstein Centre for Computational Neuroscience, University of TübingenTübingen, Germany

**Keywords:** photoreceptor, *rd* retina, spontaneous activity, gap junctions, synapse, inner retina, outer retina, visual restoration

## Abstract

During neuronal degenerative diseases, microcircuits undergo severe structural alterations, leading to remodeling of synaptic connectivity. This can be particularly well observed in the retina, where photoreceptor degeneration triggers rewiring of connections in the retina’s first synaptic layer (e.g., Strettoi et al., 2003; Haq et al., 2014), while the synaptic organization of inner retinal circuits appears to be little affected (O’Brien et al., 2014; Figures 1A,B). Remodeling of (outer) retinal circuits and diminishing light-driven activity due to the loss of functional photoreceptors lead to spontaneous activity that can be observed at different retinal levels (Figure 1C), including the retinal ganglion cells, which display rhythmic spiking activity in the degenerative retina (Margolis et al., 2008; Stasheff, 2008; Menzler and Zeck, 2011; Stasheff et al., 2011). Two networks have been suggested to drive the oscillatory activity in the degenerating retina: a network of remnant cone photoreceptors, rod bipolar cells (RBCs) and horizontal cells in the outer retina (Haq et al., 2014), and the AII amacrine cell-cone bipolar cell network in the inner retina (Borowska et al., 2011). Notably, spontaneous rhythmic activity in the inner retinal network can be triggered in the absence of synaptic remodeling in the outer retina, for example, in the healthy retina after photo-bleaching (Menzler et al., 2014). In addition, the two networks show remarkable differences in their dominant oscillation frequency range as well as in the types and numbers of involved cells (Menzler and Zeck, 2011; Haq et al., 2014). Taken together this suggests that the two networks are self-sustained and can be active independently from each other. However, it is not known if and how they modulate each other. In this mini review, we will discuss: (i) commonalities and differences between these two oscillatory networks as well as possible interaction pathways; (ii) how multiple self-sustained networks may hamper visual restoration strategies employing, for example, microelectronic implants, optogenetics or stem cells, and briefly; and (iii) how the finding of diverse (independent) networks in the degenerative retina may relate to other parts of the neurodegenerative central nervous system.

## Two Independent Oscillatory Networks in the Degenerative Retina

In the past years, spontaneous abnormal spiking activity in retinal ganglion cells has been described in several animal models of photoreceptor degeneration (Stasheff, 2008; Sekirnjak et al., 2009; Stasheff et al., 2011). Triggered by this finding, spontaneous activity in both outer and inner degenerative retina has been studied in detail (for review, see also Trenholm and Awatramani, 2015): in the outer *rd1* retina, clusters of remnant cones, rod bipolar cells (RBCs) and horizontal cells display rhythmic activity (Haq et al., 2014). In the inner *rd1* and *rd10* retina, recurrent interactions between AII amacrine cells and cone bipolar cells lead to spontaneous rhythmic spiking in retinal ganglion cells (Borowska et al., 2011; Trenholm et al., 2012). The *rd1* mouse (Bowes et al., 1990) is probably the most prominent model for retinal degeneration in Retinitis Pigmentosa in humans—despite the fact that other than in the human condition, *rd1* photoreceptor degeneration starts as early as postnatal day 10 (Paquet-Durand et al., 2011), and therefore degenerative processes likely interfere with retinal development. Nonetheless, because most research into oscillatory retinal networks has been conducted in *rd1* mouse retina, we will mainly focus in the following on this model.

Inner and outer retinal oscillatory activity shares important common features: in both cases, the cell-intrinsic mechanisms that drive oscillatory activity require spontaneous membrane potential fluctuations. In the outer retina, voltage-gated Ca^2+^ channels expressed by cone photoreceptors are essential for spontaneous activity (Haq et al., 2014), whereas in the inner retina, voltage-gated Na^+^ and K^+^ channels in AII amacrine cells play a crucial role (Borowska et al., 2011; Trenholm et al., 2012; Choi et al., 2014). Additionally, in both the inner and outer retina, glutamatergic as well as gap junction-mediated interactions (electrical synapses) are involved in spreading the activity (Margolis et al., 2014; Haq et al., 2014; Poria and Dhingra, 2015).

Despite these similarities, the activity patterns in the inner and outer retina also show a number of differences (Table 1): first and most importantly, spontaneous activity in the outer retina is likely a direct consequence of synaptic remodeling triggered by the death of photoreceptors (Phillips et al., 2010; Haq et al., 2014; for review, see Jones et al., 2010). Specifically, spontaneous activity in remnant cones, which likely drive the network, seems to be triggered by morphological changes of the cone synapse and consequentially, the lack of inhibitory feedback from horizontal cells (Haq et al., 2014). In contrast, inner retinal activity appears to result mainly from the lack of light-driven input (Menzler et al., 2014), with the gross organization of inner retinal circuits remaining largely unaltered (Borowska et al., 2011). Here, aberrant activity is likely initiated by intrinsic, degeneration-independent physiological properties of the AII amacrine cells (Cembrowski et al., 2012; Choi et al., 2014). Second, inner retinal activity comprised large networks of at least dozens of neurons (Menzler and Zeck, 2011), which is likely related to the fact that AII amacrine cells form a large electrically coupled network (reviewed in Bloomfield and Dacheux, 2001). In contrast, outer retinal activity is confined to local clusters of typically less than 10 cells (Haq et al., 2014), possible due to the sparseness of remnant cones, which appear to initiate the activity (see above). Third, the frequency of oscillations measured at the level of the cone bipolar cells, amacrine cells and ganglion cells (Margolis et al., 2008; Borowska et al., 2011) is 3–5 times higher than that of the activity recorded in cones, horizontal cells and RBCs (Haq et al., 2014). The finding that rod and cone bipolar cells show different activity patterns suggests that different sets of bipolar cell types are “recruited” by the two networks: RBCs, which make atypical synapses with cones in the *rd1* retina (Haq et al., 2014), heavily participate in the oscillatory activity in the outer retina, whereas ON and OFF cone bipolar cells play an important role in the inner retinal network by spreading the activity to the retinal ganglion cells (Borowska et al., 2011). Forth and finally, the modulatory role of synaptic inhibition is strikingly different: experiments using GABA receptor antagonists suggest that in the outer retina, GABA (presumably released from horizontal cells) reduces the frequency of oscillatory events (Haq et al., 2014). In contrast, in the inner retina, inhibition provided by GABAergic amacrine cells appears to have no effect in *rd10* (Biswas et al., 2014) or even enhances the spiking frequency of the some retinal ganglion cells in *rd1* mice (Ye and Goo, 2007). Taken together, the two oscillatory networks in the degenerative retina show remarkable structural and functional differences.

**Table 1:** **Differences between outer and inner oscillatory activity in the *rd* retina (for detailed information, see text)**.

	Outer* rd* retina	Inner* rd* retina
Degenerative re-modeling of synaptic contacts	Severe	None to minor (not enough data)
Trigger of synaptic activity	Synaptic remodeling	Intrinsic and/or lack of input from outer retina
Participating cell types	Cones, rod bipolar cells, horizontal cells (?)	Cone bipolar cells, AII amacrine cells, ganglion cells
Number of simultaneously oscillating cells	Clusters of up to 10 cells	Dozens of ganglion cells
Frequency of spontaneous events	Maximum ~3 Hz	~10 Hz
Effect of GABA receptor antagonists	Decrease in activity	Increase in activity or no effect

### Pathways from the Outer to the Inner *rd1* Retina

In the healthy retina, bipolar cells relay the photoreceptor signals to inner retinal circuits (for review, see Euler et al., 2014). Therefore it is not surprising, that in the degenerative retina, bipolar cells have been reported to participate in the spontaneous activity in both the inner and outer retina. This makes them prime candidates for a modulatory link between outer and inner activity networks during degeneration. Which are synchronously active with cones in the *rd1* retina (Haq et al., 2014), provide the main excitatory drive to AII amacrine cells in the healthy tissue (for review, see Bloomfield and Dacheux, 2001) and therefore, may play a central role also during degeneration. The axonal morphology of RBCs remains intact even when rod photoreceptor are lost (Gargini et al., 2007; Borowska et al., 2011), indicating that also the rod bipolar cell-AII amacrine cell connectivity may be preserved in the degenerating retina. Indeed, spontaneous AII amacrine cell oscillations depend on intrinsic properties of these cells instead of being an emergent property of the degenerative tissue (Choi et al., 2014), raising the question what prevents spontaneous activity in these cells in the healthy retina. One possible explanation is that the (intact) rod bipolar cell input depolarizes AII amacrine cells and thereby prevents hyperpolarization and spontaneous activity “bursts” in the AII network (Choi et al., 2014). Blocking ionotropic glutamate receptors (and thereby rod bipolar cell input) did not alter AII network activity (Borowska et al., 2011), indicating that RBcs do not drive the AII network in the *rd1* retina. However, to conclusively answer if *rd1* RBCs can relay activity from the outer to the inner retina, direct evidence is needed: a key experiment will be to functionally assess if and how the synaptic connection between RBCs and AII amacrine cells is altered during retinal degeneration.

### Pathways from the Inner to the Outer *rd1* Retina

**Figure 1:**
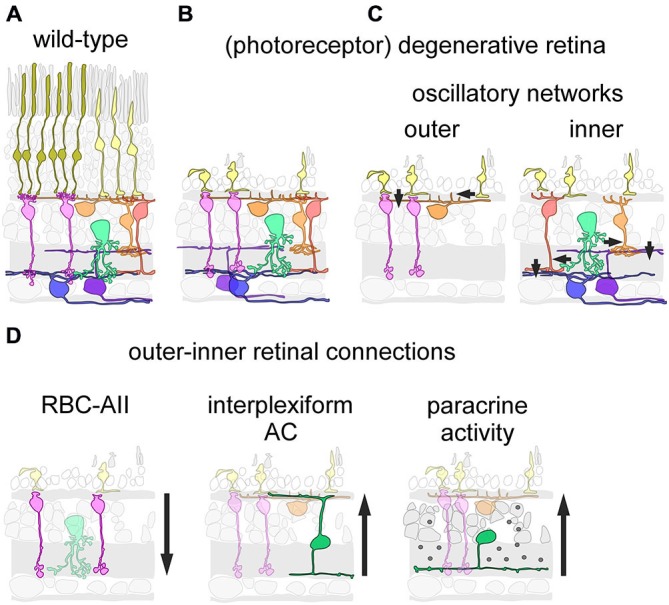
**Inner and outer oscillations in the degenerative mouse retina. (A,B)** Schematic drawing showing cellular organization in the healthy (wild-type) **(A)** and the photoreceptor-degenerative retina **(B)**. Note that in the absence of rods, some cones still persist but lack light-sensitive outer segments. **(C)** Neuronal circuits underlying outer (left) and inner (right) spontaneous activity in the degenerative retina. **(D)** Putative pathways connecting the inner to the outer retina. The rod bipolar cell (RBC) may relay outer retinal activity to the inner retina via synapses with the AII amacrine cells (AII) (left). Interplexiform amacrine cells make synaptic contacts in the outer retina, and thus could relay inner retinal activity to the outer retina (middle). Paracrine release of neuromodulators by amacrine cells could act as a “diffuse” inner-to-outer-retina pathway (right). **(C,D)** Only relevant neuron types/classes are depicted; arrows indicate direction of main signal flow.

There are three pathways that connect the inner to the outer retina and may allow the inner retinal network affecting oscillatory activity in the outer retina (Figure 1D): first, GABAergic interplexiform amacrine cells receive synaptic drive from cone bipolar cells in the inner retina and form (GABAergic) output synapses in the outer retina (Dedek et al., 2009). This GABAergic feedback pathway could modulate or even drive outer retinal activity. However, providing direct experimental evidence for this hypothesis will be difficult as the application of GABA receptor antagonists would at the same time interfere with the effects of GABA release from horizontal cells (Liu et al., 2013; Kemmler et al., 2014). Second, given the direct synaptic interaction of AIIs with dopaminergic amacrine cells (Contini and Raviola, 2003) and the strong expression of dopamine receptors on cones and horizontal cells (for review, see Mangel, 2001), it is conceivable that dopamine released in the inner retina can act on outer retinal neurons in a paracrine fashion. Many types of amacrine cells release neuromodulatory substances as a “secondary transmitter”, some of which have shown to act—like dopamine—extra-synaptically (for review, see Cervia et al., 2008; Hirasawa et al., 2015). Another, although more remote possibility may be aberrant electrical coupling of bipolar cell dendrites with cones or horizontal cells: OFF cone bipolar cells express the gap junction-forming connexins Cx36 and Cx45 on their dendrites (Feigenspan et al., 2004; Hilgen et al., 2011) and these connexins are, in principle, able to form functional gap-junctions with those expressed on cones (Feigenspan et al., 2004). In the light of the strong re-modeling of the degenerative outer retina (for review, see Jones and Marc, 2005), during which cones are “re-wired” to RBCs (Strettoi et al., 2004; Haq et al., 2014), the formation of gap junctions between cones and cone-bipolar cells cannot be excluded (for a discussion of gap junctions in neuronal remodeling, see Takeuchi and Suzumura, 2014). If these hypothesized aberrant electrical synapses were bi-directional, they may not only be able to relay inner retinal activity directly to cones but would also allow cones contribute to inner retinal activity.

Taken together, various vertical pathways may connect the oscillatory networks in one or the other direction. However, to which extent and at which stages of degeneration these interactions may play a role remains to be investigated.

## How Multiple Self-Sustained Networks may Hamper Visual Restoration Strategies

Several therapeutic approaches to treat photoreceptor degeneration or its effects on vision have been successfully tested in animal models and are close to or already in clinical trials with human patients. These treatment strategies are diverse and include stem cell technology (for review, see Mead et al., 2015), administration of growth factors (LaVail et al., 1998), tissue transplantation (for review, see Alexander et al., 2015), AVV-mediated gene therapy (for review, see Trapani et al., 2014), microelectrode implants (for review, see Zrenner, 2013), and optogenetics (Lagali et al., 2008). In contrast to the rapid advances in the development of strategies for photoreceptor restoration/replacement or stimulation of inner retinal circuits, our understanding of the functional (dis)organization in the remnant retinal circuits and its activity is still limited. More research is needed here, as the existence of two (independent) spontaneously active networks in the degenerative retina certainly impacts the success of the therapeutic strategies mentioned above, although in different ways.

Strategies that target the outer retina to specifically repair or replace photoreceptors (i.e., stem cell technologies, administration of growth factors, tissue transplantation and viral vector delivery) have to deal with the remodeled synaptic connectivity that follows photoreceptor loss (for review, see Jones and Marc, 2005) as well as with the resulting oscillatory activity. The partial restoration of visual function following virus vector-mediated gene therapy in a mouse model for autosomal recessive Retinitis Pigmentosa suggests that the synaptic organization of the outer retina remains sufficiently plastic such that when light-driven input is provided, degenerative processes can be delayed (Koch et al., 2012). However, a detailed analysis of the extent to which “correct” synaptic connections may even be restored is still lacking. For suppressing (remnant) aberrant oscillatory activity, pharmacological treatments (i.e., gap-junction blockers) have been recently discussed (Toychiev et al., 2013; Haq et al., 2014).

As outlined above, morphology and synaptic connectivity in the degenerative outer retina change far more severely than in the inner retina. Therefore, instead of trying to repair or replace photoreceptors, rendering second-order neurons (i.e., bipolar cells) light-sensitive using optogenetics (Lagali et al., 2008; Doroudchi et al., 2011) may circumvent the problem that connectivity in the outer retina may be irrevocably altered at a progressed disease state, and bipolar cells cannot be re-wired with restored photoreceptors. However, even in the case of the “light-sensing bipolar cell” approach, an essential requisite is the elimination of oscillatory activity. As an alternative to pharmacological means (see above), a combinational AVV-mediated gene therapeutic approach—that is rendering bipolar cells light sensitive and dampening spontaneous activity (i.e., by targeting AII amacrine cells and cones)—would be an elegant solution.

Our need for a better understanding of retinal circuit remodeling becomes even more pressing when considering technological solutions that are already applied in patients: light-sensitive electronic implants that stimulate the remaining retinal network electrically (Zrenner et al., 2011; Dorn et al., 2013). Some approaches aim at using the computational power of the remaining retinal circuit and therefore focus on electrically stimulating bipolar cells (Zrenner, 2013). Like the viral approach (see above) this has the additional advantage that the bipolar cell layer typically does not degenerate as profoundly as the photoreceptor layer and is still present in most patients after years of blindness (Santos et al., 1997). Clinical trials showed, however, that implant patients reported limitations, such as blurring of visual stimuli and slow recovery of sensitivity, that were not necessarily predicted from the implant’s specifications (Zrenner et al., 2011). It is conceivable that these limitations are, at least partially, due to retinal circuit rewiring and/or spontaneous oscillatory activity (Zrenner, 2013).

In any case, understanding if and how the outer and inner oscillatory networks interact at the rod bipolar cell-to-AII amacrine cell synapse is a key question, not only to understand the complexity of degenerative oscillations in the retina, but also for practical, treatment-related reasons.

## What to Learn from the Retina about Aberrant Spontaneous Activity in other Parts of the Degenerating Central Nervous System?

The spontaneous oscillations found in the degenerative retina are reminiscent of abnormal activity in other degenerative tissues of the central nervous system. For example, in animal models for epilepsy, pyramidal cells in the hippocampus show abnormal high-frequency oscillations (Foffani et al., 2007; Ibarz et al., 2010; for review, see Engel et al., 2009). Also in this system, diverse synaptic mechanisms underlying these activity patterns are currently being debated (for review, see Jefferys et al., 2012). Similar to the situation in the degenerative hippocampus in epilepsy, abnormal activity patterns in the motor circuits of the thalamo-cortical pathways are correlated with the phenotype of the Parkinson’s disease (for review, see Galvan et al., 2015). In animal models for Parkinson’s disease, diverse mechanisms located in different nuclei of the thalamo-striatal system are thought to alter neuronal activity. Although the entire complexity is not well understood, the death of dopaminergic neurons and the degeneration of dopaminergic projections along the thalamo-striatal pathways initiate remodeling of dopaminergic as well as downstream synaptic connections, accompanied by the loss of dendritic spines (Day et al., 2006), relative volume increase of the remaining spines (Villalba and Smith, 2011), increase in number of synaptic inhibitory connections (Fan et al., 2012), and reduction of dendritic arborizations (Fieblinger et al., 2014). Therefore, in some aspects this remodeling of synaptic structures of higher brain areas is reminiscent of the situation in the degenerative retina.

The retina as a well-described model system may offer a unique chance to learn more about the general mechanisms that underlie of abnormal activity in the degenerative central nervous system: its distinct degeneration-induced oscillatory networks represent an opportunity to understand whether intrinsic cellular mechanisms are sufficient to explain spontaneous activity or if remodeling of neuronal circuits is a prerequisite. Here, the outer retina would be a well-described model for assessing how synaptic remodeling leads to aberrant spontaneous activity. Complementary, the inner retina offers the chance to investigate how reduced (light-driven) synaptic input can trigger spontaneous activity in a network that is largely “normal”. Additionally, these two retinal networks will provide crucial insight into how distinct degenerative networks within the same tissue may interact with each other.

## Funding

This work was supported by the Deutsche Forschungsgeme-inschaft (EXC 307, CIN to TE and TS).

## Conflict of Interest Statement

The authors declare that the research was conducted in the absence of any commercial or financial relationships that could be construed as a potential conflict of interest.
